# OCCUPATIONAL VULNERABILITY PROFILES IN THE POLISH WORKFORCE: A NARRATIVE REVIEW OF AVEM-BASED RESEARCH

**DOI:** 10.13075/ijomeh.1896.02642

**Published:** 2025

**Authors:** Wiktor Warchałowski, Ivana Mašková, Dana Buršíková

**Affiliations:** 1 Medical University of Gdańsk, Department of Psychology, Faculty of Health Sciences with the Institute of Maritime and Tropical Medicine, Gdańsk, Poland; 2 University of West Bohemia, Department of Psychology, Faculty of Education, Plzeň, Czech Republic

**Keywords:** prevention, burnout, occupational health, vulnerability, Polish workforce, work-related patterns

## Abstract

The inventory *Work-related Coping Behavior and Experience Patterns* (*Arbeitsbezogenes Verhaltens- und Erlebensmuster* – AVEM) serves as a valuable preventive tool for the early identification of individuals at risk of burnout and occupational health issues by evaluating their work-related patterns, which may be either health-promoting or indicative of increased health risk. The aim of this narrative review was to map and synthesize research on AVEM conducted in Poland across occupational groups. A structured search of selected databases and search engines was performed, resulting in the identification of 29 sources whose findings were synthesized and compared with international evidence. The results showed that the overall proportion of Polish participants assigned to risk patterns was significantly higher than in the international context, particularly among teaching and healthcare professionals, indicating greater vulnerability to burnout and occupational health issues in these occupations. Beyond people-centered professions, women, older workers, individuals with an additional job, those living in smaller towns and villages, and those experiencing mobbing also emerged as highly vulnerable groups. These individuals also tended to be extrinsically motivated and exhibited higher levels of neuroticism, elevated stress, and poorer mental and physical health. In contrast, having a hobby and a better perceived material standing were mainly associated with healthy patterns. Individuals assigned to healthy patterns tended to be intrinsically motivated and reported higher job satisfaction, greater levels of fluid and emotional intelligence, and more adaptive personality traits. There is a clear need for targeted workplace interventions across professions in Poland to address the heightened risk of occupational health issues.

## Highlights

- Polish populations display increased vulnerability to occupational health issues.
- Professionals in people-centered occupations and women are among the most vulnerable.
- Having an additional job and living in smaller towns or villages are risk factors.
- Having a hobby and better perceived material standing are protective factors.

## INTRODUCTION

A national report published in 2024 revealed alarming findings regarding the occupational health of the Polish workforce. Nearly 80% of working Poles reported experiencing ≥1 symptom of burnout, representing a 13-percentage-point increase from approx. 65.3% reported 3 years earlier. The most commonly reported symptom was persistent fatigue or lack of energy, affecting 43.4% of respondents [1]. These findings point to a growing threat to occupational health in Poland, likely driven by global economic uncertainty and rapid technological advancements that blur the boundaries between personal and professional life, heighten employer expectations regarding constant availability, and limit opportunities for recovery. Since these challenges are more likely to intensify than diminish in the near future, the focus of practitioners and researchers should shift toward preventive measures – particularly the early identification of individuals who may be especially vulnerable to developing occupational health issues, followed by targeted interventions to enhance their resilience to work-related stress. In this context, the concept of work-related coping behavior and experience patterns, together with its corresponding inventory, serves as a valuable preventive tool for assessing typical work-related behavior and experience patterns that may signal vulnerability to burnout and other occupational health issues, especially when problematic patterns persist without timely intervention [2]. The *Arbeitsbezogenes Verhaltens- und Erlebensmuster* (AVEM) inventory [3], which has also been referred to as the *Work-related Coping Behavior and Experience Patterns* (WCEP) [4,5], the *Measure of Coping Capacity* (MECCA) [6,7], or the *Occupational Stress and Coping Inventory* [8] in some English-language publications, assesses 11 dimensions across 3 areas.

These include:

– professional commitment (subjective significance of work, professional ambition, tendency to exert, striving for perfection, emotional distancing);
– coping capacity (resignation tendencies, offensive coping with problems, balance and mental stability);
– subjective well-being (satisfaction with work, satisfaction with life, experience of social support).

Specific work-related patterns – validated across multiple studies and offering valuable insights for targeted occupational health interventions – are identified by analyzing scores across these dimensions and assigning individuals to 1 of 4 profiles derived through cluster analysis. Two profiles (G and S) are health-promoting, while 2 (A and B) indicate health risks:

– healthy ambitious (G): high ambition, balanced emotional distancing, strong coping skills, and well-being. These individuals achieve quality work without compromising leisure;
– unambitious (S): low professional commitment but adequate coping and well-being, indicating no health risks but low motivation;
– excessively ambitious (A): high work significance and exertion, low emotional distancing, weak coping, and poor well-being. This profile resembles workaholism and increases risks for occupational health issues, particularly cardiovascular issues;
– resigned (B): low commitment, ineffective coping (e.g., resignation), and poor well-being. This profile resembles late-stage burnout symptoms and increases risks for occupational health issues, burnout, and psychosomatic issues in particular [2,3].

These pattern descriptions refer to fully developed prototypes with >95% similarity to the respective reference profile. However, such clear cases are relatively rare. According to Schaarschmidt and Fischer [2], 5 levels of pattern expression can be distinguished:

– full (similarity >95%),
– accentuated (>80% and ≤95%),
– tendential (>50% and ≤80%, no second pattern >30%),
– combined (2 patterns >80%, the weaker pattern >30%),
– non-classifiable (no clear assignment).

The AVEM questionnaire was adapted to Polish cultural conditions and validated on a sample of 616 workers in various helping professions. The Polish version of the AVEM questionnaire, labeled *Kwestionariusz do badania indywidualnych wzorców zachowań i przeżyć związanych z pracą*, showed good psychometric qualities [9]. Since the Polish adaptation of the AVEM instrument became available, a growing number of empirical studies have applied it across diverse professional groups in Poland. However, these studies remain dispersed across various fields and are less accessible, as many have been published in lower-visibility sources, such as national journals not indexed in major international databases or institutional book chapters. As a result, there is a lack of consolidated knowledge on how AVEM patterns are distributed in the Polish workforce, or how they relate to occupational, psychological, and health-related variables. This fragmentation limits both the theoretical integration and the practical application of the findings. The aim of this review is to identify existing Polish research in a structured way, synthesize it, and ultimately integrate it into the broader body of international AVEM research. The review adopts a narrative approach based on structured database and manual searches, with the goal of mapping and synthesizing findings on AVEM patterns across occupational groups in Poland, drawing on all available empirical sources that report AVEM distribution and/or AVEM correlates, without limiting the scope to a specific time frame. Accordingly, the research question underpinning the review was formulated as follows: What insights does existing AVEM research provide regarding AVEM distribution across occupational groups in Poland, and what are the key correlates that act as risk or protective factors influencing (un)healthy work-related patterns? By bringing together findings that have remained scattered, the review aims to reveal broader trends, highlight country-specific risk and protective factors, and enhance the potential of AVEM-based research to inform occupational health policy and targeted interventions in the Polish context.

## METHODS

### Eligibility criteria

The review includes all literature that utilized the AVEM inventory in the Polish workforce and reported the distribution of the 4 AVEM patterns described in [3] and/or their correlates. Studies in English or Polish were considered, while those that did not assign participants to the AVEM pattern typology were excluded. The authors included studies using all above-described levels of AVEM pattern expression, as long as a primary classification into the 4 AVEM patterns (G, S, A, B) was provided.

### Search strategy

To identify relevant literature, key terms related to the AVEM instrument were combined with terms referencing the Polish workforce and searched in databases (Web of Science and Scopus) and the Google Scholar search engine. The Boolean search string used in the database queries was as follows: (“Arbeitsbezogenes Verhaltens- und Erlebensmuster” OR “work-related coping behavior and experience patterns” OR “AVEM” OR “WCEP” OR “wzorce zachowań i przeżyć związanych z pracą”) AND (“Polish” OR “Poland” OR “Polska” OR “kwestionariusz”).

No date restrictions were applied during the search; all available studies meeting the inclusion criteria were considered regardless of publication year. The searches were conducted between June 27 and September 13, 2024, with the upper limit of the inclusion period reflecting the time at which the review was conducted.

### Study selection process

**Figure 1.**
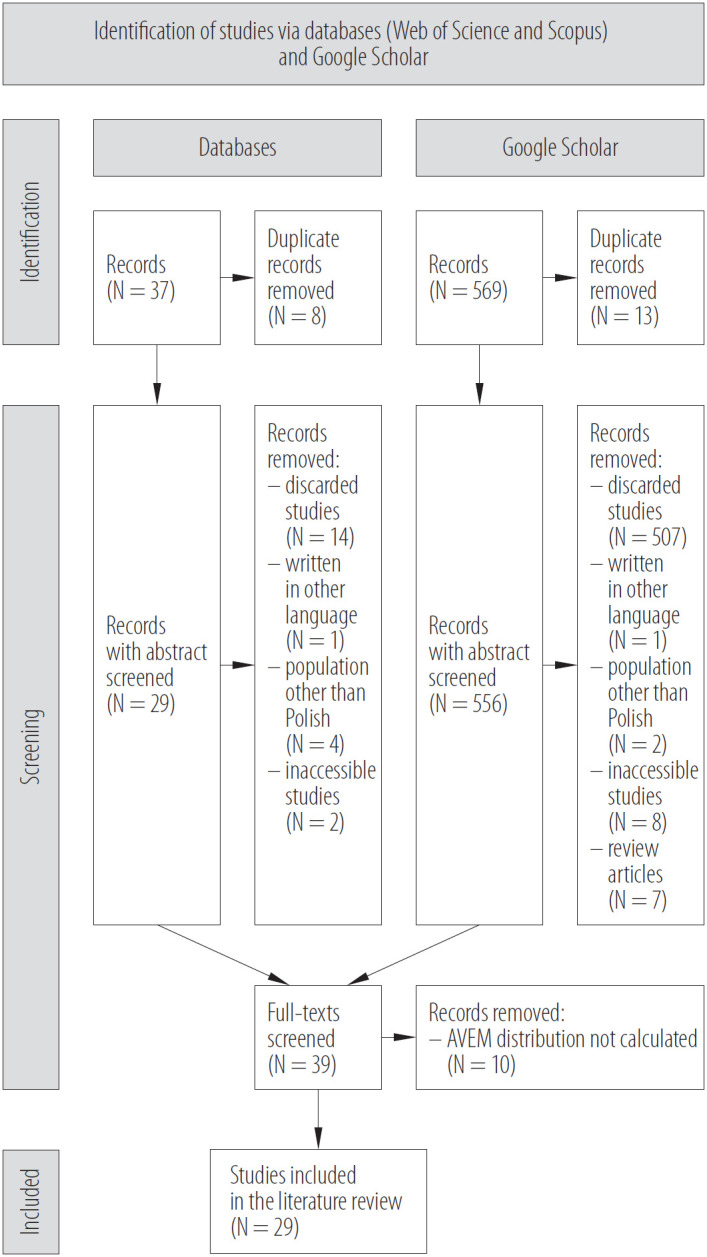
A flowchart of the search strategy of the studies on the early identification of individuals at risk of burnout and occupational health issues published 2007–2024 and included in the review, search conducted June–September 2024

Adapted from PRISMA Statement [10].

The database search identified 37 articles, and Google Scholar returned 569 records. Abstracts were manually screened for relevance, ensuring studies were accessible, utilized the AVEM questionnaire, were not review articles, focused on the Polish workforce, and were written in Polish or English. Duplicates were also removed during this process, excluding 29 records from the database search and 538 from the Google Scholar search. Subsequently, full texts were analyzed to confirm the inclusion of AVEM pattern typology calculations and/or investigations of pattern correlates. This step excluded an additional 10 studies, resulting in a final total of 29 works. Refer to Figure 1 for a flowchart illustrating the literature selection process. Detailed data from the included records are provided in Table 1.

**Table 1.** A summarised overview of the studies on the early identification of individuals at risk of burnout and occupational health issues published 2007–2024 and included in the review, search conducted June–September 2024

Reference	Data collection period	Sample characteristics	Pattern distribution	Findings on AVEM correlates
Aouil et al. [11,12]	n.a.	employees in social services, education, administration, healthcare, and other sectors (N = 1000, 76.3% females, age M±SD 40.67±9.84 years)	– full sample: G = 30%, S = 22%, A = 24%, B = 24% – social services: G = 24%, S = 6%, A = 48%, B = 22% – educational sector: G = 27%, S = 28%, A = 26%, B = 19% – administration: G = 37%, S = 19%, A = 26%, B = 18% – healthcare: G = 29%, S = 24%, A = 19%, B = 28% – other sectors: G = 30%, S = 15%, A = 32%, B = 23%	– gender: women (↑ A/B) – seeking psychological help (↑ G/S/A, ↓ B) – prior psychological consultations (↑ G/A) – having an additional job (↑ A/B) – having a hobby (↑ G, ↓ B) – length of professional experience (↑ G/S) – marital status, income, age (G = S = B = A)
Bartosiewicz et al. [13]	May 25–June 24, 2021	primary and secondary school teachers (N = 412, 65.38% females, age M±SD 41.7±8.36 years)	G = 11.62%, S = 7.75%, A = 32.20%, B = 48.43%	– self-efficacy (↑ G/A, ↓ S/B) – job satisfaction (G > A > B > S) – age: <37 years (↑ G/A, ↓ S/B), 38–47 years (↑ B, ↓ G) – length of work experience: <5 years (↑ G, ↓ B), 5–15 years (↑ B, ↓ G/S/A) – position: certified teacher (↑ B, ↓ G), contract teacher (↑ A, ↓ S/B), trainee teacher (↑ B, ↓ G), appointed teacher (↑ G, ↓ A/B)
Bartosiewicz and Łuszczki [14]	beginning of 2022	nurses (N = 795, 100% females, age M±SD 40.89±10.47 years)	G = 11.19%, S = 11.19%, A = 30.19%, B = 47.43%	– work experience: 6–10 years compared to 1–5 years (↑ G) – education: master's degree compared to secondary education (↑ G) – place of work: private sector and primary health care compared to hospital (↓ S), outpatient specialist care compared to hospital (↑ A, ↓ S) – having an additional training course (↑ A) – career anchors: leadership (↓ S), challenge (↑ G) – job satisfaction (↑ G, ↓ B) – career anchors: security and stability, lifestyle, service and commitment to others (G = S = A = B)
Basińska et al. [15]	n.a.	soldiers (N = 141, 0% females, age M±SD 33±10 years)	G = 32.9%, S = 28.3%, A = 23.7%, B = 15.0%	emotional intelligence (↑ G, ↓ B)
Basińska and Andruszkiewicz [16]	n.a.	nurses working in hospital (N = 150, 100% females, age M±SD 40.11±7.16 years)	G = 9.8%, S = 31.8%, A = 16.4%, B = 42.0%	– positive thinking (G/A > B) – direct action (G/S/A > B) – seeking help (G > B)
Basińska and Dreas [17]	n.a.	military officers (N = 112, 0% females, age M±SD 33.7±10.4 years)	n.a.	– neuroticism (↑ A/B, ↓ G) – extraversion (↑ G, ↓ S/A/B) – openness (↑ G, ↓ B) – agreeableness (↑ S, ↓ A/B) – conscientiousness (↑ G, ↓ S/B)
Basińska et al. [18]	2006–2007	nurses (N = 331, 100% females, age M±SD 34.15±6.61 years)	G = 25%, S = 16%, A = 31%, B = 28%	– manageability (↑ G/S, ↓ B) – comprehensibility (↑ G/S, ↓ B) – meaningfulness (↑ G/S, ↓ A/B) – sense of coherence (↑ G/S, ↓ A/B)
Betke et al. [19]	November 2015–June 2016	nurses (N = 150, 100% females, age M±SD 41.34±8.82 years)	G = 10%, S = 22%, A = 2.67%, B = 15.33% non-classifiable 50%	– somatic symptoms (↑ A) – anxiety and insomnia (↓ G) – symptoms of depression (↑ B) – general mental health (↓ G) – social functioning disorders (G = S = A = B)
Drozd [20]	June 2020–April 2021	social workers (N = 150, 87.6% females, age M±SD 42.52±9.72 years)	G = 18%, S = 30%, A = 2.67%, B = 49.33%	emotional exhaustion, depersonalization, and personal accomplishment (G = S = A = B)
Góralewska-Słońska [21]	2nd half of 2017	employed full-time and part-time students of psychology, management, logistics, economics and management and production engineering (N = 180, 68.3% females, age M±SD 27.30±7.79 years)	G = 24%, S = 27%, A = 23%, B = 26%	– mobbing (↑ B, ↓ G) – masculinity (↑ G, ↓ B) – femininity (↑ B, ↓ S)
Haor et al. [22]	November–December 2012	nurses (N = 100, 100% females)	G = 26%, S = 16%, A = 22%, B = 36%	n.a.
Horoszkiewicz [23]	2008–2009	drivers (N = 1022, 2.94% females)	G = 61%, S = 12%, A = 23%, B = 4%	n.a.
Horoszkiewicz and Korchut [24]	n.a.	drivers (N = 188, 0% females, age M±SD 37.7±11.3 years)	G = 58.39%, S = 13.18%, A = 24.14%, B = 4.85%	– fluid intelligence (↑ S, ↓ A) – age (↑ A, ↓ S)
Jachimowicz-Wołoszynek et al. [25]	March–June 2009	nurses (N = 106, 100% females, age M±SD 40±7 years)	G = 16%, S = 22%, A = 21%, B = 41%	hospital wards (G = S = A = B)
Karabanowicz [26]	n.a.	teachers of public and special schools (N = 80, 93.75% females, age M±SD 42.38±8.83 years)	G = 0%, S = 6.2%, A = 3.8%, B = 90%	– task-oriented coping style (↓ A) – emotion-oriented coping style (↑ S, ↓ B) – avoidant style, distraction, seeking social contact, type of school (G = S = A = B)
Krajnik et al. [27], Muszalska et al. [7]	January 2005–June 2006	palliative care specialists (N = 79) and other medical practitioners (N = 223, 33% surgeons, 38% general practitioners, 29% anaesthetists)	– palliative care specialists: G = 30%, S = 14%, A = 38%, B = 18% – surgeons: G = 27%, S = 11%, A = 47%, B = 15% – general practitioners: G = 20%, S = 16%, A = 40%, B = 24% – anaesthetists: G = 25%, S = 15%, A = 25%, B = 35%	– mental health (↑ G/S [palliative specialists only]) – neuroticism (↑ B, ↓ G/S) – extraversion (↑ G, ↓ B) – openness (↑ G, ↓ B) – conscientiousness (↑ G, ↓ B) – agreeableness (G = S = A = B)
Mariańczyk and Otrębski [29]	n.a.	individuals with disabilities enrolled in a job preparation course (N = 46, 41.3% females)	G = 13%, S = 5%, A = 3%, B = 5%, non-classifiable = 75%	– social adaptation (↑ G, ↓ A/B)
Mroczek et al. [30,31]	2015–2016	medical workers (N = 432, N_AVEM_ = 424, 33.49% physicians, 37.73% nurses, 28.77% paramedics, 69% females, age M±SD 33.6±11.7 years)	– full sample: G = 31.8%, S = 19.1%, A = 24.1%, B = 25% – paramedics: G = 45.9%, S = 22.1%, A = 18.0%, B = 13.9% – nurses: G = 28.1%, S = 21.3%, A = 21.9%, B = 28.8% – physicians: G = 23.9%, S = 14.1%, A = 37.7%, B = 30.3%	– gender: women compared to men (↓ S) – level of general social competence (↑ G) – level of competence in intimate situations (↓ B) – age; years in the profession; years in the previous job (G = S = A = B)
Mróz [32]	n.a.	nurses (N = 183, 100% females, age M±SD 43.5±5.97 years)	G = 19%, S = 11%, A = 43%, B = 27%	– overall resilience (↑ G, ↓ B) – determination (↑ G, ↓ B) – openness and humor (↑ G, ↓ B) – coping competence (↑ G/S, ↓ B) – failure tolerance (↑ G/S, ↓ B) – optimism (↑ G/S, ↓ B) – total perceived stress (↑ B, ↓ G) – emotional tension (↑ B, ↓ G/S) – intrapsychic stress (↑ B, ↓ G) – external stress (↑ B, ↓ G)
Napora et al. [9]	n.a.	nurses (N = 372, 50% single mothers, 100% females, age M±SD 44.49±7.96 years)	– single mothers: G = 15%, S = 26%, A = 21%, B = 39% – mothers from 2-parent families: G = 22%, S = 24%, A = 20%, B = 34%	– help-seeking strategy (↑ G) – avoidance/resignation strategy (↓ S [single mothers only]) – work satisfaction (↑ G, ↓ B [entire sample], ↓ S [single mothers only])
Olkiewicz and Andruszkiewicz [33]	n.a.	neurological nurses (N = 50, 98%, age M±SD 35.82±6.99 years)	G = 12%, S = 30%, A = 17%, B = 41%	– educational level (↓ S) – material standing (↑ G) – job satisfaction (↑ G, ↓ B) – religious belief (↑ G) – place of residence: smaller towns/villages (↑ B)
Olszewski [34]	n.a.	special school teachers (N = 100, 92% females, age M±SD 43.65±10.46 years)	G = 35%, S = 24%, A = 22%, B = 16%	motives for work in the beginning: – G: top 3: M5, M17, M16; bottom 3: M2, M7, M9 – S: top 3: M5, M17, M16; bottom 3: M7, M4, M9 – A: top 3: M5, M16, M15; bottom 3: M2, M9, M7 – B: top 3: M5, M17, M7; bottom 3: M11, M4, M9 motives to continue work: – G: top 3: M5, M16, M17; bottom 3: M13, M7, M2 – S: top 3: M17, M5, M15; bottom 3: M4, M2, M7 – A: top 3: M14, M16, M5; bottom 3: M13, M2, M7 – B: top 3: M5, M6, M15; bottom 3: M9, M2, M4 – M9 (↑ G) – M2 (↑ B) – external factors (↑ A/B)
Ronginska and Doliński [35,36]	2014–2016	mid-level managers in public companies (N = 2164)	G/S/A = n.a., B = 9.15%	n.a.
Skonieczna-Żydecka et al. [37]	December 2014–January 2015	paramedics (N = 31.0% females)	G = 48.4%, S = 25.8%, A = 12.9%, B = 12.9%	sensory integration disorder (↑ A, ↓ G)
Zawadzka [38]	2015–2019	prison staff in their second year of preparatory service (N = 102, 27.5% females)	G = 62%, S = 34%, A = 3%, B = 1%	n.a.

n.a. – not available.

N_AVEM_ – number of participants out of the entire sample for whom the pattern assignment is available.

The sample characteristics refer to the entire sample and may slightly deviate from the characteristics of the subsample for which the pattern assignment is available. Only correlates with a clearly defined association with the pattern assignment were included in the overview. Totals of pattern distribution may deviate from 100% due to rounding imprecisions.

Codes for motives for work: M2 – no motive/accidental choice, M4 – the desire to achieve or maintain a certain social position, M5 – willingness to work with people who need help, M6 – convenient working hours, M7 – the need for a career change, M9 – unwillingness to change profession, M11 – rewarding salary, M13 – family tradition, M14 – creative nature of work, M15 – pursuing an interesting career, M16 – interest in disability, M17 – a passion for work involving direct contact with others.

### Narrative thematic synthesis

To organize the findings across studies, the authors used a narrative thematic approach in which categories were developed inductively. During the data extraction process, recurring topics and correlates of AVEM patterns were identified and grouped into 15 thematic domains to enable a structured synthesis of results. These thematic domains included:

– profession,
– gender,
– age,
– partnership and personal life,
– education,
– socioeconomic characteristics,
– length of professional experience,
– type of workplace and work environment,
– job satisfaction,
– work motivation,
– intelligence,
– personality traits,
– adaptive characteristics,
– stress and coping strategies,
– mental and physical health.

## RESULTS

The 29 studies included in the review were published in 2007–2024, reflecting the range of results from the unrestricted search. Across the included studies, a generally consistent approach to AVEM pattern classification was observed. Most studies applied a method based on the identification of the most dominant AVEM pattern, which corresponds to the tendential classification level (similarity >50%) and reflects the default procedure provided by the AVEM authors and implemented in the standardized scoring algorithm [2]. This method was either explicitly stated or could be inferred from the reported data. Only 2 studies diverged from this approach: Betke et al. [19] classified participants only when the probability of assignment exceeded 80%, corresponding to the accentuated and full levels, while Mariańczyk and Otrębski [28] used an even stricter threshold of >95%, equivalent to full expression only. As a result, both studies reported a number of non-classifiable individuals whose profiles did not meet the defined cut-off. The reviewed research on AVEM has primarily focused on Polish professionals in people-oriented occupations, such as medical staff, social workers, and teachers [11–14,22,29–31]. Additional studies have examined soldiers [15,17], police officers [19], students [21], drivers [23,24], individuals with disabilities [28], managers [34,35], and prison staff in preparatory service [37]. The following sections summarize the findings of AVEM research within the Polish workforce.

### Profession

The distribution of work-related patterns varies significantly across occupations. People-oriented professions – such as teaching, social work, and healthcare – exhibit a notably higher prevalence of risk patterns A and B. For example, up to 93.8% of teachers displayed these risk patterns [26], alongside 52–70% of social workers [12,20]. Within the healthcare sector, the proportion of nurses exhibiting risk patterns ranged from 50.7% [29,30] to 77.62% [14]. Among physicians, the prevalence ranged from 56% [28] to 68% [29,30]. In contrast, paramedics demonstrated the lowest tendency toward risk patterns among medical professionals, with up to 74.8% classified under healthy work patterns [36]. Occupations such as professional driving, service work, and administrative roles also exhibit a more favorable distribution of work-related patterns. For instance, 62% of prison staff [37] and 61% of drivers [23] were assigned to the healthy pattern G. Moreover, only 9% of managers were classified under the least desirable risk pattern B [34,35].

### Gender

Research indicates significant gender differences in the distribution of work-related patterns. Among medical staff, men are more frequently assigned to pattern S [29,30], whereas women in various professions tend to be assigned more often to patterns A or B [11]. Similarly, research on students from various academic disciplines revealed a positive association between femininity and pattern B, as well as a negative association between femininity and pattern S. Conversely, masculinity was positively linked to pattern G and negatively linked to pattern B. These findings suggest that femininity may be a risk factor for unhealthy patterns, whereas masculinity may serve as a protective factor [21].

### Age

The relationship between work-related patterns and age remains inconclusive, though some evidence suggests a growing tendency toward risk pattern assignment with increasing age. Studies have found no significant association between age and AVEM pattern among professionals in education, social work, public administration, or healthcare [11,29,30]. Among teachers, younger individuals were more frequently assigned to patterns G and A, both indicative of strong professional motivation, while older teachers showed a greater propensity for pattern B [13]. Similarly, in the case of drivers, the likelihood of assignment to pattern S declined with age, whereas the probability of exhibiting pattern A increased [24].

### Partnership and personal life

The association between partnership and work-related patterns is less straightforward. Aouil et al. [11] found no significant differences in pattern distribution based on marital status among individuals employed in various sectors. In contrast, research [9] suggests that relationship status may influence work-related patterns among nurses who are mothers, as single mothers were slightly more likely to be classified under risk patterns compared to their counterparts in 2-parent households. Additionally, engaging in a hobby was associated with a greater likelihood of being assigned to pattern G and served as a protective factor against pattern B [11].

### Education

Research on nurses has also provided insights into the relationship between work-related patterns and education. In Poland, nurses can attain 1 of 3 educational levels: secondary medical education, a bachelor's degree, or a master's degree in nursing [38]. Bartosiewicz and Łuszczki [14] found that nurses with a master's degree were more likely to be assigned to pattern G. In contrast, lower educational levels were associated with a higher likelihood of being assigned to pattern S [32]. Additionally, completing further training courses, such as a nursing specialization course, was linked to a greater tendency toward pattern A [14].

### Socioeconomic characteristics

There appears to be a relationship between work-related patterns and socioeconomic factors, such as personal income, having an additional job, and place of residence. Although no association was found between work-related patterns and the nominal value of income among professionals from various occupations [11], a positive perception of one's own material standing among nurses was associated with an increased likelihood of pattern G [32]. In contrast, having an additional job was linked to an increased tendency toward risk patterns among individuals in various occupations [11]. Regarding place of residence, nurses living in smaller towns or villages were more frequently assigned to pattern B compared to those living in larger cities [32].

### Length of professional experience

Length of professional experience, or the number of years in a profession, plays a role in explaining pattern distribution, though the relationship is not linear and varies by profession. Although among medical professionals, neither years in the profession nor years in the previous job differentiated between patterns [29,30], research on individuals in various professions indicates that more experienced workers are generally less vulnerable, with a higher proportion classified into healthy patterns [11]. Similarly, a moderate length of professional experience (6–10 years) was associated with a higher likelihood of being assigned to pattern G in nurses compared to their early-career counterparts [14]. In contrast, early-career teachers exhibited the healthiest pattern distribution, which tended to become less favorable as their careers progressed. In addition, different professional statuses reflecting the level of seniority were associated with varying distributions of work-related patterns. Appointed teachers most frequently displayed pattern G, contract teachers tended toward pattern A, while both trainee and certified teachers showed an increased tendency toward pattern B [13].

### Type of workplace and work environment

The specific type of workplace appears to influence work-related patterns. Although no statistically significant differences were found in pattern distribution among teachers working at different types of schools [26] or among nurses in different hospital wards [25], working in a hospital increased the likelihood of being assigned to pattern S, while employment in outpatient specialist care was associated with pattern A [14]. An adverse working environment – especially mobbing, defined as persistent negative communicative actions directed at an individual – negatively affects pattern distribution. Specifically, Góralewska-Słońska [21] found that experiencing mobbing decreases the likelihood of being assigned to pattern G and increases the likelihood of being assigned to pattern B.

### Job satisfaction

Research has clearly confirmed that job satisfaction, among nurses and teachers, is associated with pattern G and serves as a protective factor against pattern B [9,13,14,32]. Among nurses who were single mothers, job satisfaction also protected against pattern S [9].

### Work motivation

Research on the link between work-related patterns and work motivation was conducted with Polish special school teachers. Olszewski [33] found that while certain motivations are common among teachers assigned to different patterns – such as cognitive, personal, and ideological motives at the beginning of their careers, including a desire to help people, interest in disability, and a passion for working with others – motives evolve over time and differ across patterns. Teachers assigned to pattern G are more likely to be motivated by a long-term commitment to their careers. In contrast, teachers assigned to pattern S are primarily motivated by a competitive salary, which remains a significant factor throughout their careers. For these individuals, passion for working with people and salary are key motivators for staying in the profession. Teachers assigned to pattern A, at the start of their careers, are driven by external factors like workplace proximity or salary. Later, their motivation shifts toward attaining social rank, pursuing creative work, and opportunities for knowledge acquisition and skill development. On the other hand, teachers assigned to pattern B are primarily motivated by external factors such as workplace convenience or flexible hours. Notably, 38% of teachers assigned to pattern B reported that their initial decision to pursue a teaching career was accidental [33]. A different perspective is offered by Bartosiewicz and Łuszczki [14], who investigated career anchors – i.e., personal values and motivations guiding individuals' career decisions – among nurses. They found that while career anchors related to security and stability, lifestyle, and service and commitment to others did not differentiate among work-related patterns, pattern S was associated with a lower level of the leadership career anchor, and pattern G was linked to a higher level of the challenge career anchor.

### Intelligence

Available research has also explored the relationship between work-related patterns and intelligence, specifically fluid intelligence and emotional intelligence. Higher levels of fluid intelligence, which reflect an individual's cognitive abilities, were associated with pattern S, while lower levels were linked to pattern A in professional drivers [24]. The potential link between emotional intelligence – defined as the ability to understand one's own and others' emotions – and work-related patterns was examined in professional soldiers. This study found that higher levels of emotional intelligence were associated with pattern G, whereas lower levels were linked to pattern B [15].

### Personality traits

The relationship between work-related patterns and personality was examined using the Big 5 personality traits model, which defines personality in terms of extraversion, conscientiousness, openness, agreeableness, and neuroticism. In both military officers and medical staff, pattern G was associated with higher levels of extraversion, openness, and conscientiousness, as well as lower levels of neuroticism. Lower levels of neuroticism were also observed in pattern S. Both patterns S and A were linked to lower levels of extraversion, with pattern A additionally showing higher levels of neuroticism and lower levels of agreeableness. Lastly, pattern B was associated with higher levels of neuroticism and agreeableness, and lower levels of extraversion, openness, and conscientiousness [7,17].

### Adaptive characteristics

Research has also explored the association between work-related patterns and several adaptive characteristics, including sense of coherence, religious belief, resilience, self-efficacy, and social adjustment. Specifically, teachers assigned to patterns G and A tended to exhibit higher self-efficacy, whereas those assigned to patterns S and B displayed lower or average levels of self-efficacy [13]. Sense of coherence and its dimensions were positively associated with healthy patterns G and S, and negatively associated with risk patterns A and B in nurses [18]. Religious belief was also positively correlated with the healthy pattern G [32]. With respect to resilience, pattern G was positively related to determination, openness, coping competence, failure tolerance, and optimism, whereas pattern B was negatively linked to these characteristics. Pattern S was positively associated with coping competence, failure tolerance, and optimism [31]. Regarding social adjustment, research on individuals with disabilities enrolled in a job preparation course found that individuals assigned to pattern G were better adjusted than those in patterns A and B [28]. Finally, it was revealed that social skills play a role in pattern distribution: whereas pattern G was linked to an increased level of general social competence, pattern B was linked to a decreased level of competence in intimate situations among medical workers [29,30].

### Stress and coping strategies

The level of stress experienced by individuals and the coping strategies they use in dealing with such stress significantly differentiate among work-related patterns. In nurses, general stress levels – as well as levels of emotional tension, intrapsychic stress, and external stress – were higher in those assigned to pattern B and lower in those assigned to pattern G. Additionally, decreased emotional tension was observed in nurses assigned to pattern S [31]. With respect to coping strategies, positive thinking, direct action, and help-seeking were positively linked with pattern G and negatively linked with pattern B. In addition, positive thinking and direct action were also higher in pattern A, and direct action in pattern S [9,16]. Furthermore, in nurses who were single mothers, pattern S was linked to a decreased tendency toward the avoidance/resignation strategy [9]. In teachers, pattern S was associated with an increased tendency to adopt emotion-oriented coping, whereas in pattern B, this tendency was decreased. Pattern A, on the other hand, was linked to a decreased tendency to adopt task-oriented coping. Other coping styles – such as avoidant style, distraction, and seeking social contact – did not differentiate among the patterns [26].

### Mental and physical health

Research shows that there is a clear link between aspects of mental and physical health and work-related patterns. Specifically, in nurses, higher scores on the *General Health Questionnaire* (GHQ-28), indicating poorer mental health, were associated with a decreased tendency toward pattern G. Additionally, pattern B was linked to symptoms of depression, while pattern A was associated with somatic symptoms. In contrast, individuals assigned to pattern G were less likely to experience anxiety and insomnia [19]. Among palliative care specialists, better mental health was associated with the healthy patterns G and S [27]. Nevertheless, symptoms of burnout were not associated with pattern distribution in social workers [20]. An interesting finding reported by Aouil et al. [11] is that individuals from various professions assigned to patterns G and A were more likely to have had prior psychological consultations, whereas those assigned to pattern B were less inclined to seek psychological support, such as consulting a mental health professional. With respect to physical health, Skonieczna-Żydecka et al. [36] found that paramedics assigned to pattern G exhibited fewer symptoms of sensory processing disorder compared to paramedics assigned to pattern A.

## CONCLUSIONS

The aim of this review was to identify empirical studies that have employed the AVEM inventory specifically within the Polish workforce and to synthesize findings related to the distribution of the 4 work-related patterns – pattern G (healthy ambitious), pattern S (unambitious), pattern A (excessively ambitious), and pattern B (resigned) – as well as their correlates. A total of 29 studies published in 2007–2024 were identified and analyzed.

To situate the integrated findings from Polish AVEM-related research within the broader body of empirical evidence – primarily derived from German-speaking populations – the authors adopt a comparative perspective aimed at identifying key similarities and differences across contexts. To better understand the key findings, the authors interpret them through relevant theoretical frameworks drawn from occupational and health psychology. Consistent with international results [3], Polish professionals in people-centered occupations – especially in teaching and healthcare – appear especially vulnerable to burnout and other occupational health issues, as indicated by the high prevalence of risk patterns A and B. However, a notable difference emerges when Polish data are compared with German findings: the proportion of individuals assigned to the risk patterns is significantly higher in the Polish context. For example, even among the most vulnerable group, teachers, approx. 60% of German teachers fell into the risk patterns [3], whereas in several Polish samples, the proportion reached 80% [13] or even exceeded 90% [26]. This discrepancy also aligns with earlier AVEM research [39], which noted a strong tendency toward the risk patterns in teachers in former Eastern Bloc regions, including Poland. A similar trend is observed among nurses: while 42% of German nurses were assigned to the risk patterns [3], the corresponding proportion in Polish samples ranged from 50.7% [29,30] to as high as 77.62% [14]. Strikingly, these alarmingly high rates of vulnerability are not observed across all occupational groups in Poland. In sharp contrast to the situation of teachers and nurses, >70% of Polish drivers [23,24] and >90% of prison staff [37] displayed healthy patterns. This profession-specific contrast suggests that not all sectors are equally affected and points to deeper structural and occupational drivers of these differences. An interpretative framework is offered by the social determinants of health (SDH) theory, which suggests that health inequalities are socially produced and that individual health outcomes are shaped by broader socioeconomic and cultural conditions [40]. The disproportionately high vulnerability observed among Polish teachers and healthcare professionals, compared to both their German counterparts and other Polish occupational groups, may reflect long-standing structural disadvantages affecting public-sector professions in post-socialist contexts – such as chronic underinvestment, low pay, precarious job conditions, and policy inconsistency. These broader disadvantages are also reflected in everyday working conditions, which can be further understood through the job demand–control–support (JDCS) model. This model suggests that vulnerability increases when high job demands are combined with low autonomy and insufficient social support [41]. From this perspective, Polish teachers face growing parental and societal expectations, an increasing bureaucratic workload driven by regulatory changes, and limited autonomy due to strict oversight by local education authorities [42,43]. A similar situation affects Polish nurses, who manage demanding workloads resulting from staff shortages and have little professional independence, as their role typically involves following physicians' orders rather than making autonomous decisions [44,45]. These occupational profiles reflect the high-strain work environments described by the JDCS model and illustrate how broader structural conditions and everyday working conditions combine to increase the risk of adverse occupational health outcomes.

Further insights come from studies examining individual and psychosocial correlates of the AVEM patterns. The well-documented trend of increased vulnerability among women has been confirmed in Polish samples, where both being female and possessing feminine traits were associated with the risk patterns A and B. In contrast, being male and displaying masculine traits were linked to the healthier patterns G and S [11,21,29,30]. This finding is consistent with evidence from university student populations in German-speaking countries [46] as well as among German professionals across various occupational fields [3], and aligns with broader research on gender disparities in mental health [47].

The relationship between age, length of professional experience, and work-related patterns appears to be complex and not entirely consistent. While research suggests a growing tendency toward risk pattern assignment with increasing age, the association between pattern distribution and length of professional experience is not linear and varies by profession. Findings in nurses and teachers – that shorter and moderate durations of work experience have been linked to the healthy pattern G [13,14] – partially support a core assumption of the AVEM model, developed within the teacher occupational health framework: that pattern distribution is generally more favorable at the beginning of a professional career and tends to deteriorate over time [39]. However, the ambiguous relationship between age, work experience, and AVEM patterns is characteristic not only of the Polish studies included in this review but also of research from Germany [48]. These findings suggest that the influence of age and experience – along with their potential interaction – may be profession-specific rather than generalizable across occupational groups.

Polish research has not fully confirmed the findings from the German population, where marital status has been shown to have a protective effect against risk patterns [6], partly due to a lack of available studies. Nevertheless, Polish studies offer a novel finding: a link between having a hobby and an increased tendency toward pattern G as well as a decreased tendency toward pattern B [11]. This is largely consistent with existing evidence on the health-promoting effects of leisure activities [49] and can also be explained by the job demands–resources (JD-R) model, which emphasizes that personal resources – such as engaging in restorative hobbies – help buffer the effects of high job demands and lower burnout risk [50].

A potentially country-specific finding concerns the educational level of nurses. While education level had no significant effect on pattern assignment in a mixed German and Austrian sample [48], Polish data show that higher education was associated with the healthy pattern G, lower education with the unambitious pattern S, and participation in additional specialization courses with the excessively ambitious risk pattern A [14,32]. One possible explanation for this unique finding comes from self-determination theory (SDT) [51], which underscores the role of autonomous motivation in health and well-being. The empirical link between pattern G and autonomous motivation has been confirmed in prior research [52]. In this light, higher education may reflect greater autonomous motivation linked to pattern G, whereas lower education may be linked to reduced autonomous motivation, consistent with the low-engagement characteristics of pattern S. Conversely, participation in specialization courses may involve predominantly controlled motivation, driven by institutional requirements or external pressures. When such expectations are not matched by adequate rewards or recognition, they can foster overcommitment and stress, aligning with the excessively ambitious risk pattern A. Given Poland's specific regulations regarding nursing education, these dynamics may not be directly comparable internationally, yet they provide valuable insights for policymakers aiming to strengthen nurse training and professional development.

With regard to personal income, Polish research indicates that while nominal income level had no significant effect on pattern distribution [11], a higher subjective assessment of one's material standing was associated with a greater likelihood of exhibiting the healthy pattern G [32]. This finding aligns with research from a German-speaking sample [48], which linked pattern G to the perceived importance of having a high income. Together, these results suggest that subjective perceptions of income adequacy may be more relevant than nominal income, as the latter fails to reflect individual variations in living costs. This interpretation is further supported by studies on mental health and burnout, which show that satisfaction with one's income can buffer against negative psychological outcomes [53].

The finding that living in villages and smaller towns is associated with the resigned pattern B, while residence in larger cities is linked to the healthy pattern G [32], is unique within the existing AVEM literature. This result may be partly related to subjective income adequacy, as rural living often involves higher transportation costs, which could reduce perceived financial comfort. Additionally, longer commuting times may reduce leisure time, contributing to increased strain. Another possible explanation comes from mental health research, which highlights structural barriers – such as limited access to mental health services and greater stigma around seeking psychological help – as factors that may negatively impact the mental health of rural residents [54]. This interpretation also aligns with the SDH framework [41], which emphasizes that health outcomes are shaped by broader socioeconomic and structural conditions, including geographic and infrastructural inequalities. Another important finding that has not yet been supported by international AVEM research is that having an additional job increases the likelihood of being assigned to the risk patterns A and B [11]. This finding is generally in line with evidence on the link between multiple jobholding and burnout [55]. Since in Poland the weekly hours spent in a second job are almost 2.5 times higher than in Germany [56], having an additional job may be an important factor in partially explaining the observed cross-country differences in pattern distribution among German and Polish professionals. Given that having a hobby is also a protective factor against pattern B [11], lack of time caused by multiple jobholding may contribute to the increased vulnerability in the Polish workforce also through reduced engagement in restorative leisure activities.

The findings regarding the specific type of workplace that differentiates work-related patterns among nurses align with AVEM research conducted in German samples [57,58]. This suggests that professionals within the same occupational group may exhibit varying levels of vulnerability, depending on differences in job content, responsibilities, and working conditions. This interpretation is further supported by the observed impact of the work environment. In this context, the Polish research emphasized mobbing – a form of workplace bullying. Similar to findings in the Polish sample [21], bullying in the German sample was associated with a higher likelihood of pattern B and a lower likelihood of pattern G [4]. These findings are consistent with both national and international evidence on the detrimental effects of workplace bullying on mental health [59,60].

Another important factor distinguishing the most optimal, healthy pattern G from the resigned pattern B was job satisfaction, which consistently emerged as a protective factor against pattern B in both Polish [9,14,32] and German research [61,62]. With regard to work motivation, available evidence comes mainly from a Polish study on special school teachers, which found that teachers assigned to pattern G were primarily driven by intrinsic motivation (e.g., long-term commitment to the profession), while teachers assigned to patterns S and A showed mixed intrinsic and extrinsic motives. In contrast, teachers assigned to pattern B were mostly driven by extrinsic motives, with over one-third reporting that their decision to enter the teaching profession was accidental [33]. This Polish research complements AVEM-based findings from German studies on teacher education students. Specifically, students assigned to pattern B reported the lowest level of intrinsic motivation for pursuing a teaching career and were more likely to choose teacher education due to its low difficulty or as a fallback solution due to a lack of alternatives or personal confidence [5,63]. These findings align with SDT [51], which links intrinsic motivation to greater engagement and well-being. Taken together, this body of evidence highlights a practitioner-relevant concern: individuals assigned to pattern B tend to demonstrate problematic professional motivation that may warrant early attention in career guidance and training programs.

Polish AVEM-related literature also provides unique findings regarding fluid and emotional intelligence. Higher levels of fluid intelligence were associated with pattern S, whereas lower levels were linked to pattern A – an important pioneering finding in the AVEM literature [24]. This finding corresponds with existing evidence on the association between IQ and mental health [64]. With respect to emotional intelligence, which was found to be increased in pattern G and decreased in pattern B [15], a similar result was reported by Hofmann et al. [65], who observed higher levels of emotion regulation in pattern G and lower levels in pattern B among German teachers. This body of evidence aligns with research highlighting the strong protective influence of emotional intelligence against burnout [66].

With respect to Big 5 personality traits, the results are in accordance with available AVEM-related evidence in that individuals assigned to risk patterns exhibited higher levels of neuroticism compared to those assigned to healthy patterns. In contrast, higher levels of extraversion, openness, and conscientiousness were specifically linked to pattern G [7,5,17], and agreeableness to pattern S [5,17]. Results related to other personal characteristics seem to mirror findings available in the German literature, with minor specifics. The link between healthy patterns and increased sense of coherence [18] mirrors the findings among Egyptian university lecturers [67], and the increased religious belief in pattern G [33] aligns with the religious/spiritual orientation observed in German pastors [68]. The healthy patterns were also linked to resilience, both in Polish nurses [31] and German teacher education students [3]. The observed link between pattern G and better social adjustment [28], and between pattern B and poor social skills [29,30], may be supported by findings on psychosocial competence in German teacher education students assigned to pattern G [69]. A unique result, only partially aligning with evidence from international literature, was the association between work-related patterns and self-efficacy. Whereas in Polish teachers increased self-efficacy was linked to patterns G and A [13], in German teacher education students it was related to patterns G and S [70], suggesting a possible cultural moderation in how self-beliefs influence AVEM patterns.

Another surprising finding among Polish nurses was the increased use of adaptive coping strategies in both G and A patterns, and their decreased use in S and B patterns. This contrasts with findings from Germany, where functional coping was linked to the healthy patterns G and S, and dysfunctional coping to patterns A and B, with pattern A in particular associated with elevated use of strategies such as alcohol consumption and smoking [71,72]. An equally unexpected result was observed in Polish teachers: a higher tendency toward emotion-oriented coping in pattern S and a lower tendency in pattern B [26]. This finding is atypical, as a Swiss study involving teacher education students reported the opposite – emotion-oriented coping was more prevalent in pattern B and less so in the healthy patterns [73]. These differences suggest that AVEM patterns are shaped by both personal characteristics and the broader cultural and occupational context in which individuals work.

Finally, findings from the Polish workforce regarding stress levels and mental and physical health align closely with international AVEM-related research. One study found clear differences in stress levels between nurses assigned to patterns G and B [31]. This aligns with international studies involving students and professionals from various fields, which consistently show that risk patterns are associated with higher stress levels, while healthy patterns offer protection against both general and job-specific stressors [46,74]. Similarly, research on mental and physical health indicates that risk patterns are linked to a range of issues, such as depression and somatic symptoms [19], as well as sensory processing disorder symptoms [36]. In contrast, healthy patterns are associated with lower incidences of such symptoms, including anxiety and insomnia [19]. These findings not only support the theoretical foundations of the AVEM typology but are also consistent with international evidence demonstrating that healthy patterns correlate with better mental and physical health, whereas risk patterns are associated with health problems [46,62]. An additional noteworthy finding [11] is that individuals with the resigned pattern B – marked by low engagement and a sense of limited control – are, ironically, less likely to seek psychological help, a tendency consistent with the learned helplessness framework [75]. This highlights an important challenge for occupational health prevention, as those who might benefit most from support may be the least likely to access it.

This study identified and analyzed empirical findings on work-related coping behavior and experience patterns within the Polish workforce. By integrating these findings, it contributes meaningfully to the international body of research on this concept. In particular, it expands the existing evidence – previously based largely on German-speaking samples – by placing it in a broader international context, thereby enhancing its generalizability. The study's comprehensive focus on the Polish workforce also offers valuable insights for shaping national occupational health policy. Drawing from the integrated results and their comparison with international evidence, the following conclusions are presented.

The overall proportion of Polish participants assigned to risk patterns was significantly higher than in the international context, particularly among teaching and healthcare professionals, indicating greater vulnerability to burnout and occupational health issues in these occupations. Beyond people-centered professions, women, older workers, individuals with an additional job, those living in smaller towns and villages, and those experiencing mobbing also emerged as highly vulnerable groups. These individuals also tended to be extrinsically motivated and exhibited higher levels of neuroticism, elevated stress, and poorer mental and physical health. In contrast, having a hobby and a better perceived material standing were mainly associated with the healthy patterns. Individuals classified under the healthy patterns tended to be intrinsically motivated and reported higher job satisfaction, greater levels of fluid and emotional intelligence, and more adaptive personality traits.

Several studies have contributed pioneering findings within the AVEM literature, such as the associations between work-related patterns and having a hobby or an additional job, place of residence, fluid and emotional intelligence, and the tendency to seek psychological help – although these findings require international validation to be fully contextualized. While the research findings generally align with international AVEM-related studies, several unique results emerged that warrant further investigation. Notably, these include the unexpected association of pattern A with increased self-efficacy and adaptive coping strategies, as well as a reversed tendency in pattern distribution linked to emotion-oriented coping.

### Limitations

The main limitation of this study lies in the relatively small number of available publications, each of which focuses on a distinct population and examines a relatively unique set of AVEM-related variables. This heterogeneity leads to a fragmented body of evidence, making cross-study comparisons difficult. Consequently, the findings are less easily aligned with international literature, as it remains unclear whether the observed effects are specific to the individual samples studied, reflective of broader national or occupation-specific trends, or indicative of country-specific characteristics. This lack of clarity limits the generalizability of the results and underscores the need for more empirical evidence on this topic within the Polish context. A further limitation is that the studies primarily used a cross-sectional design, which cannot detect causal effects. Therefore, the factors associated with the work-related patterns should be interpreted as correlates rather than direct predictors or causes. Further, the review includes literature of varying quality and also incorporates some sources that have not yet undergone peer review, such as preprints, which should be interpreted with appropriate caution. Another limitation is that no weighting or formal quality appraisal of the included studies was performed. This was due to substantial heterogeneity in study populations, methodologies, and reported outcomes, as well as the lack of standardized effect measures. As this is a narrative review, the findings were synthesized thematically rather than statistically. It should also be noted that while the AVEM classification approach was consistent across most studies – typically assigning participants to the most dominant pattern – 2 studies deviated by applying stricter thresholds for classification. This difference in assignment criteria should be considered when interpreting cross-study comparisons of AVEM type distribution. Finally, it should be noted that the list of included studies may not be comprehensive. While major academic databases such as Scopus and Web of Science were searched systematically, many relevant sources had to be located via Google Scholar, which does not allow precise search strings and therefore required extensive manual screening – an approach that may lack the same level of systematicity as database searches. Additionally, several sources – primarily theses – were excluded due to the unavailability of full texts.

### Practical implications

The findings of this review point to a pressing need for profession-specific interventions in Poland, aimed at reducing the elevated risk of burnout and related occupational health problems – particularly in the most vulnerable sectors. Below, the authors outline several actionable recommendations grounded in the synthesized findings of AVEM studies conducted in Poland.

Given the alarming prevalence of vulnerable individuals in teaching and healthcare professions, preventive measures should begin as early as higher education. The authors recommend that the AVEM inventory be administered in teacher education, medical, and nursing programs as a self-reflective and preventive tool to help students identify vulnerabilities at an early stage. Preventive intervention modules could be integrated into curricula to provide guidance on coping strategies and resilience building. For timely correction of risk patterns, effective programs already exist – such as Strengthened for the Teaching Profession [76] – which could be adapted to the Polish context.

Employers should prioritize creating a healthy and safe working environment in which employees' basic psychological needs – such as autonomy, competence, and relatedness – are met. This includes fostering mutual trust, enabling independent decision-making, encouraging constructive communication, and maintaining a positive workplace climate that actively prevents bullying and other forms of mistreatment.

Employers and policymakers should strive to improve working conditions for all workers, including those in the public sector. Excessive workloads – whether stemming from administrative burdens or chronic staff shortages – should be addressed through structural solutions. Setting realistic performance expectations, coupled with ensuring fair and adequate wages, would help maintain a healthy work–life balance and allow individuals to cover their living expenses without resorting to additional employment. Such measures could reduce the risk of overburden and overcommitment, thereby lowering the likelihood of developing risk work-related patterns.

Psychological counseling and intervention programs should be made accessible to all occupational groups to reduce work-related stress and strengthen individual coping capacity. Evidence shows that mainly cognitive-behavioral and mindfulness-based interventions – including web-based formats – can effectively improve mental health and well-being [77]. Such measures should be made particularly accessible to at-risk groups as identified in AVEM research, including professionals in people-centered occupations, women, older workers, and individuals living in rural areas.
